# Baicalin Alleviate Apoptosis via PKC-MAPK Pathway in Porcine Peritoneal Mesothelial Cells Induced by *Glaesserella parasuis*

**DOI:** 10.3390/molecules27165083

**Published:** 2022-08-10

**Authors:** Qirong Lu, Lang Zhou, Ziyue Wang, Xiaomin Li, Li Ding, Yinsheng Qiu, Pu Guo, Chun Ye, Shulin Fu, Zhongyuan Wu, Yu Liu

**Affiliations:** Hubei Key Laboratory of Animal Nutrition and Feed Science, School of Animal Science and Nutritional Engineering, Wuhan Polytechnic University, Wuhan 430023, China

**Keywords:** *Glaesserella parasuis*, apoptosis, baicalin, porcine peritoneal mesothelial cells, PKC-MAPK

## Abstract

*Glaesserella parasuis* (GPS), a causative agent of Glässer’s disease, is thought to be the main fatal cause of peritonitis in swine, thus resulting in high mortality and morbidity and significant economic losses to the swine industry. However, the mechanisms of GPS infection-induced apoptosis and possible therapeutic pathway for GPS infection in peritonitis remain unclear. Baicalin has important biological functions during disease treatment, such as antiviral, bacterial inhibition, anti-apoptosis, and anti-inflammatory. However, whether baicalin has anti-apoptotic effects during the process of GPS infection in peritonitis is unclear. In the present study, the anti-apoptotic effect and mechanisms of baicalin in GPS infection-induced apoptosis were investigated in porcine peritoneal mesothelial cells (PPMC). The results showed that baicalin could inhibit the apoptosis rate occurrence of PPMC induced by GPS to various degrees and inhibit the expression of apoptosis-related genes and cleaved caspase-3. Meanwhile, baicalin significantly antagonized the expression of p-JNK, p-p38, and p-ERK induced by GPS in PPMC. These findings for the first time demonstrate that baicalin exerted the effect of antagonizing GPS induced apoptosis in PPMC by inhibiting the activation of the PKC-MAPK pathway and could be a therapeutic option in the management of GPS infection.

## 1. Introduction

*Glaesserella parasuis* (GPS), a gram-negative bacterium, is an early commensal of the upper respiratory tract of weaned piglets, which causes high mortality and morbidity and significant economic losses to the swine industry [1,2]. The overall prevalence of GPS in pigs in China was 27.8%, and the prevalence of GPS disease in nursing piglets (29.2%) was higher than in other pig ages [1]. Glässer’s disease due to GPS infection is characterized clinically by a systemic inflammatory response with predominant fibrinous polyserositis, peritonitis, meningitis, and polyarthritis, leading to death in swine due to sepsis during severe infections [3,4], and often present a mortality rate of 5–10% [5]. The current mainstay of prevention and treatment for Glässer’s disease is vaccine prophylaxis versus antimicrobial therapy [5]. However, antimicrobials can only kill bacterial and cannot eliminate the cell damage caused by GPS, and antimicrobials also cause problems such as veterinary drug residues, environmental pollution, and elevated drug resistance [5,6,7,8]. Therefore, seeking ways to effectively control GPS is much needed in current pig production.

Peritonitis is a common clinical symptom of GPS infection [4,9,10] and may stem from the impairment of inflammation-induced apoptosis [11]. Meanwhile, numerous studies have shown that the effective treatment of peritonitis is closely related to the regulation of apoptosis [12,13,14]. As reported, apoptosis inhibitor of macrophage ameliorates fungal induced peritonitis injury in mice [14], and short-term regulation of apoptosis may help accelerate recovery after peritonitis and prevent irreversible peritoneal damage [15]. An anti-inflammatory and apoptotic regulator, the alpha-1 antitrypsin protein, could inhibit formaldehyde-induced apoptosis of human peritoneal mesothelial cells via a caspase-mediated pathway [13]. Moreover, acetylation of high mobility group box-1 (HMGB1) by the JNK1 signaling pathway was observed to be able to promote lipopolysaccharide induced apoptosis via elevating the expression of Bax and cleaved-caspase 3 in peritoneal mesothelial cells in a peritonitis model [16]. The above studies indicated that the control of GPS induced apoptosis may serve as an effective control pathway to reduce the peritonitis damage induced by GPS.

Baicalin, one of the major bioactive flavonoids extracted from the roots of Scutellaria baicalensis Georgi, has been clinically used in humans and animals for its antimicrobial, anti-apoptosis, anti-inflammatory, antitumor, antiviral, and antioxidant properties [17,18,19]. Baicalin acts poor solubility (160 µg/mL) and low oral bioavailability (2.2% after oral administration to rats). Our previous study on the pharmacokinetics of sodium baicalin found that an intramuscular injection of sodium baicalin in piglets resulted in rapid and complete absorption, with a mean maximal plasma concentration of 77.28 ± 7.40 µg/mL at 0.17 hr and a high absolute bioavailability of 83.73 ± 5.53% [20]. Meanwhile, our previous study also showed that baicalin can inhibit the apoptosis and inflammation of porcine peripheral monocytes and porcine vascular endothelial cells induced by baicalin, and the inhibitory mechanism may be related to the inhibition of the PKC-MAPK signaling pathway [21,22,23]. However, to our knowledge, whether baicalin regulates the PKC-MAPK signaling pathway and then participates in alleviating GPS induced porcine peritoneal mesothelial cells (PPMC) has not been reported.

As mentioned earlier, GPS is considered a serious adverse factor for peritonitis in pigs. However, the exact mechanism by which GPS causes peritonitis in pigs remains to be explored, and effective chemicals for the treatment of injury caused by GPS induced peritonitis also remain to be studied. Therefore, in this study, we investigated for the first time whether baicalin can effectively participate in the PKC-MAPK pathway in the treatment of GPS induced peritonitis injury from the perspectives of baicalin, GPS, and PPMC. This may help to identify baicalin as a novel therapeutic agent for GPS to protect pigs from Glässer’s disease induced peritonitis injury.

## 2. Results

### 2.1. Baicalin Inhibits GPS Induced Apoptosis

**Figure 1:**
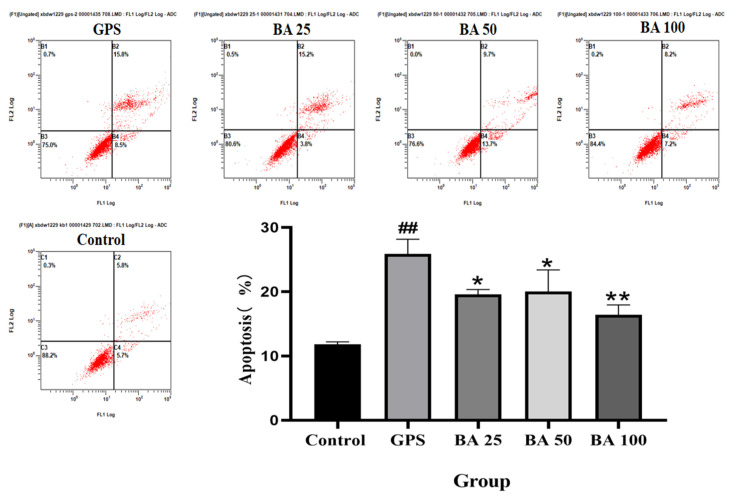
Effects of baicalin on GPS induced apoptosis in PPMC (Mean ± SD, *n* = 3). GPS: *G. parasuis* group, BA 25: 25 mg/kg baicalin + *G. parasuis* group, BA 50: 50 mg/kg baicalin + *G. parasuis* group, BA 100: 100 mg/kg baicalin + *G. parasuis* group. ^##^ *p* < 0.01 vs control. * *p* < 0.05 vs *G. parasuis* group, and ** *p* < 0.01 vs *G. parasuis* group.

To investigate whether baicalin could inhibit GPS induced apoptosis, flow cytometry was applied to detect the occurrence of apoptosis rate. As shown in Figure 1, differences in apoptosis appeared in each group after PPMC induction by GPS. Compared with the control group, the GPS model group showed significantly more apoptotic cells and an extremely high apoptotic rate; When compared with the GPS model group, 25 and 50 μg/mL baicalin significantly decreased the apoptosis rate, and 100 μg/mL baicalin could significantly reduce the number of apoptotic cells and decrease the apoptotic rate. The above study showed that 25–100 μg/mL baicalin could inhibit the apoptosis rate occurrence of PPMC induced by GPS to various degrees.

### 2.2. Baicalin Inhibits the Expression of Apoptosis-Related Genes Induced by GPS

**Figure 2:**
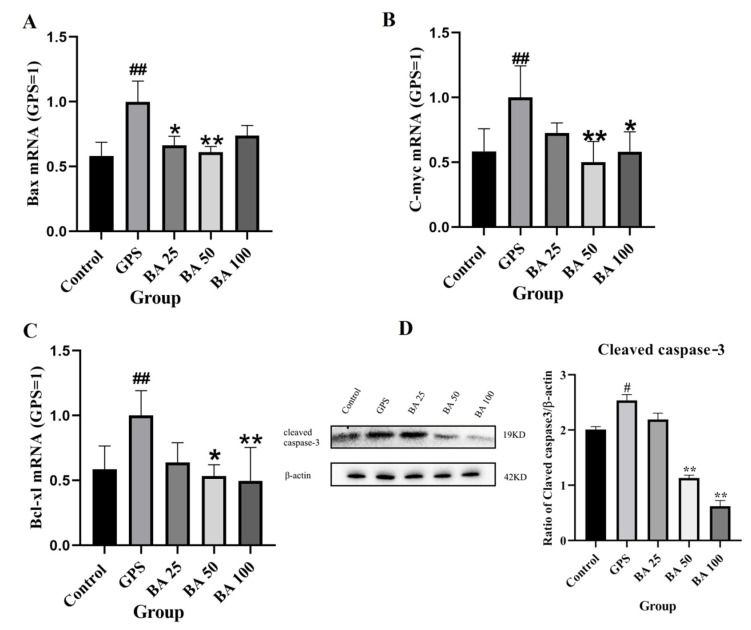
Effects of baicalin on gene expression of Bax (**A**), C-myc (**B)**, Bcl-xl (**C**), and protein expression of cleaved caspase-3 (**D**) in PPMC induced by GPS. GPS: *G. parasuis* group, BA 25: 25 mg/kg baicalin + *G. parasuis* group, BA 50: 50 mg/kg baicalin + *G. parasuis* group, BA 100: 100 mg/kg baicalin + *G. parasuis* group. ^#,##^ *p* < 0.01 vs control. * *p* < 0.05 vs *G. parasuis* group, and ** *p* < 0.01 vs *G. parasuis* group.

To explore the mechanism by which baicalin inhibits GPS induced apoptosis, RT-qPCR and western blot were applied to explore the effects of baicalin on differential genes of GPS induced apoptosis. As shown in Figure 2A–C, after the GPS treatment of PPMC, the expressions of Bax, C-myc, and Bcl-xl gene in GPS model group was significantly higher than that in the control group; 25 μg/mL baicalin inhibit the expression of C-myc and Bcl-xl gene in cells to a certain extent; and 50 μg/mL and 100 μg/mL baicalin could significantly inhibit the expression of Bax, C-myc, and Bcl-xl gene in cells. In addition, the protein expression of cleaved caspase-3 was greatly elevated in the GPS model group compared with the control group; 25 μg/mL baicalin group showed no significant difference in the amount of cleaved caspase-3 compared with the GPS model group. However, 50 μg/mL and 100 μg/mL baicalin both significantly inhibited the protein expression of cleaved caspase-3 in cells (Figure 2D). The above studies showed that 50 μg/mL and 100 μg/mL baicalin all inhibited GPS induced apoptosis of PPMC to various degrees.

### 2.3. Baicalin Inhibits GPS Induced Apoptosis via PKC-MAPK Pathway

**Figure 3:**
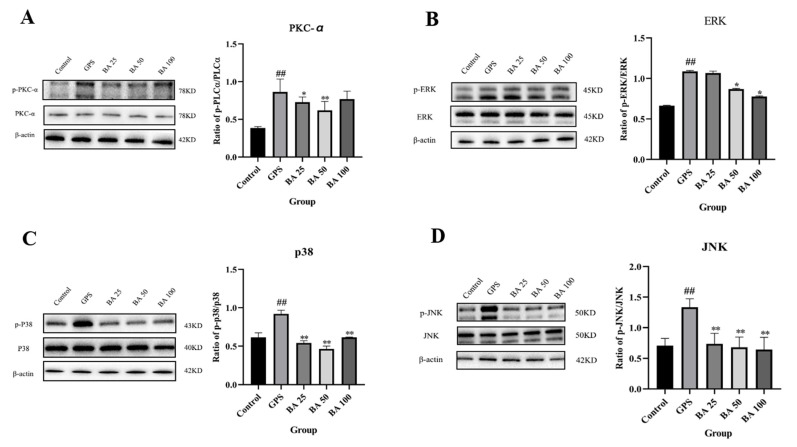
Inhibition effects of baicalin on PKC/MAPK signaling pathway in PMCC activated by GPS. PKC-α (**A**), ERK (**B**), p38 (**C)**, JNK (**D**) (Mean ± SD, *n* = 3). GPS: *G. parasuis* group, BA 25: 25 mg/kg baicalin + *G. parasuis* group, BA 50: 50 mg/kg baicalin + *G. parasuis* group, BA 100: 100 mg/kg baicalin + *G. parasuis* group. ^##^ *p* < 0.01 vs control. * *p* < 0.05 vs *G. parasuis* group, and ** *p* < 0.01 vs *G. parasuis* group.

**Figure 4:**
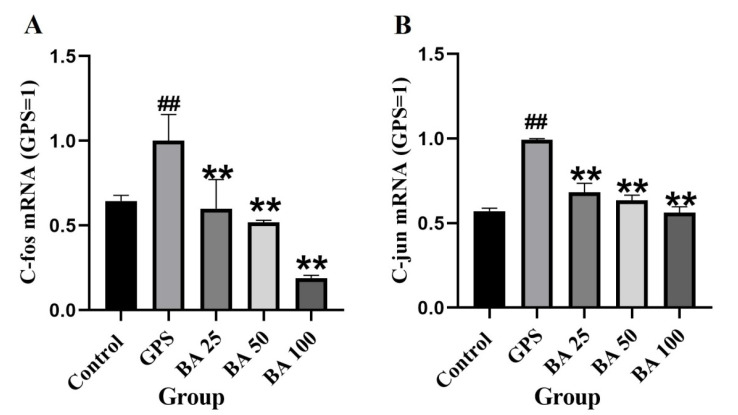
Effects of baicalin on gene expression of c-fos (**A**) and c-jun (**B**) in PMCC activated by GPS (Mean ± SD, *n* = 3). GPS: *G. parasuis* group, BA 25: 25 mg/kg baicalin + *G. parasuis* group, BA 50: 50 mg/kg baicalin + *G. parasuis* group, BA 100: 100 mg/kg baicalin + *G. parasuis* group. ^##^ *p* < 0.01 vs control. ** *p* < 0.01 vs *G. parasuis* group.

Some studies have shown that PKC-MAPK has a close link with the occurrence of apoptosis [21,24]. Therefore, in this study, we investigated whether baicalin could regulate the PKC-MAPK pathway to antagonize the occurrence of GPS induced apoptosis. As shown in Figure 3A–D, compared with the control group, the GSP group could significantly induce the expression of p-PKCα protein. However, treatment with 25 and 50 μg/mL baicalin could counteract the expression of p-PKCα induced by GPS to various degrees. Moreover, PPMC treated with GPS significantly induced the expression of p-JNK, p-p38, as well as p-ERK protein. However, 25–100 μg/mL baicalin significantly antagonized the expression of p-JNK and p-p38 induced by GPS (Figure 3C,D). Concentration of baicalin in 50 and 100 μg/mL significantly antagonized GPS induced p-ERK expression (Figure 3B). PPMC treated with GPS significantly induced the mRNA expression of c-fos and c-jun. However, 25–100 μg/mL baicalin significantly antagonized the mRNA expression of c-fos and c-jun induced by GPS (Figure 4). The above studies demonstrated that baicalin exerted the effect of antagonizing GPS induced apoptosis in PPMC by inhibiting the activation of the PKC-MAPK pathway.

## 3. Discussion

As reported, Glässer’s disease caused by GPS infection seriously endangers the pig farming industry and is found all over the world and causes severe economic losses due to high morbidity and mortality [25,26]. The overall prevalence of GPS in pigs in China was 27.8%, and the prevalence of GPS disease in nursing piglets (29.2%) was higher than in other pig ages [1], and often present a mortality rate of 5–10% [26]. Peritonitis is one of the main clinical manifestations of Glässer’s disease [4,23]. However, the pathogenic factors that contribute or trigger the onset of this disease are still unknown. Therefore, it is necessary to explore the mechanism by which GPS infection causes peritonitis, and possibly find key chemical and targets for the prevention and treatment of peritonitis. Fortunately, in this study, we discovered that baicalin interferes with the PKC-MAPK pathway and has the potential to treat the inflammation-induced apoptosis of peritonitis caused by GPS infection in PPMC cells, suggesting that baicalin has the potential to be a candidate drug for the treatment of peritonitis caused by GPS infection.

The pathogenesis of peritonitis is often accompanied by apoptosis, as reported in several studies. Severe peritonitis is often accompanied by apoptosis and inflammatory cell accumulation [12,27], and apoptosis of inflammatory macrophages and neutrophils often occurs concomitantly during acute peritonitis [28]. Sepsis caused by peritonitis is thought to be an uncontrolled regulation of the inflammatory response, leading to extensive apoptosis of lymphocytes [29], and immune protection against infectious peritonitis in endotoxin challenged mice is associated with reduced neutrophil apoptosis [30]. Moreover, apoptosis inhibitor of macrophage ameliorates fungal induced peritonitis injury in mice [14], and short-term regulation of apoptosis may help accelerate recovery after peritonitis and prevent irreversible peritoneal damage [15]. Furthermore, AP-1, one transcriptional activator within the cell, is a heterodimer composed of c-fos and c-jun. AP-1 determines the life-or-death fate of cells according to extracellular stimuli, and the activation of AP-1 is associated with a large number of apoptotic scenarios [31].

The above studies means that the control of peritonitis induced apoptosis is also a key regulatory means to treat or alleviate peritonitis. Meanwhile, the protein encoded by Bcl-xl belongs to the Bcl-2 protein family. Bcl-2 protein family members form heterodimers with Bax to participate in a variety of cellular activities as anti-apoptotic regulators [32]. Based on the results of the present study, it can be seen that GPS infection also could significantly induce the apoptosis of PPMC, and baicalin has the ability to alleviate the apoptosis of PPMC induced by GPS infection, implying that baicalin has the potential to be an adjuvant treatment for GPS infection induced peritonitis.

PKC-MAPK could be used as a pathway for the treatment of inflammation-induced apoptosis of peritonitis caused by GPS infection. Emerging evidence suggests that PKC-MAPK has a close relationship with apoptosis. For example, pretreatment of the cells with a selective PKC inhibitor significantly inhibited the apoptosis of leukemia cells induced by the combination of lactacystin/bryostatin 1 [33]. Angiotensin II induces myocardial apoptosis by activating the PKC-MAPK signaling pathway [34]. JNK activation through the PKC pathway may be one of the important mechanisms that 12-O-Tetradecanoyl-phorbol-13-acetate induces apoptosis of gastric cancer cells [35]. In norcantharidin induced human melanoma A375-S2 cell, MAPK-dependent apoptosis was mediated by PKC activation [36], through activating the JNK and p38 MAPK pathways. Moreover, the previous study also demonstrated that GPS could induce apoptosis via the PKC-MAPK pathway in mononuclear phagocytes of piglet, and baicalin could inhibit the activation of the PKC-MAPK signaling pathway through down-regulating p-JNK, p-p38, p-ERK, and p-PKC-α in mononuclear phagocytes of a piglet triggered by GPS [21]. However, whether baicalin could modulate apoptosis via the PKC-MAPK signaling pathway in PPMC during GPS infection has not been investigated. Hence, in the present study, baicalin could counteract the expression of p-PKCα induced by GPS to various degrees, and baicalin significantly antagonized the expression of p-JNK, p-p38, and p-ERK induced by GPS and the mRNA expression of c-fos and c-jun induced by GPS. These results showed that baicalin exerted the effect of antagonizing GPS induced apoptosis in PPMC by down-regulating the expression level of the apoptotic genes and inhibiting the activation of the PKC-MAPK pathway. However, the distribution of baicalin in the body is the focus of our subsequent in vivo study. Meanwhile, GPS infected pigs often exhibit multiple disease patterns and baicalin acts as a potential drug to antagonize GPS infection; this implies a need for further mining of the key disease targets of baicalin in GPS infection models as well as the in-depth mechanisms, which can help to design more effective drugs based on GPS infection targets and mechanisms and combine the maternal structure of baicalin to achieve precise treatment of diseases and optimal therapeutic effects.

## 4. Materials and Methods

The study was carried out at Animal Experimental Base in Sinopharm Animal Health Corporation Ltd. (Wuhan, China). All experimental protocols agreed with the Wuhan Polytechnic University Laboratory Animals Welfare and Animal Experimental Ethical Inspection (reference number WP20100501).

### 4.1. Drugs

Baicalin was provided by National Institutes for Food and Drug Control (B110715-201318, Beijing, China).

### 4.2. Bacterial Strains

*G.parasuis* strain SH0165 serovar 5 was used, which was isolated from the lung of a commercially produced pig with the typical characteristics of Glässer’s disease. The SH0165 isolate was cultured at 37 °C for 12 h in tryptic soy broth (Difco, Lawrence, KS, USA) or grown for 24 h in tryptic soy agar (Difco, Lawrence, KS, USA) supplemented with nicotinamide adenine dinucleotide (Sigma, St. Louis, MO, USA) and fetal bovine serum (Gibco, Gaithersburg, MD, USA).

### 4.3. Cell Culture

A piglet weighing about 10 kg, and pentobarbital sodium solution was injected intramuscularly behind the ear for about 10–15 min of anesthesia. After complete anesthesia, the piglet was sacrificed by bloodletting. Alcohol cotton was used to disinfect the abdomen of piglet. A scalpel was used to slice the skin, muscle layer, and fat layer vertically nearby the abdominal midline, find the peritoneum, and strip the fat tissue around the peritoneum. The removed peritoneal tissue was placed in PBS solution at 4 °C. The cryopreserved peritoneum was removed within six hours; peritoneal tissue was rinsed repeatedly with PBS at room temperature until blood none. Trypsin was added in a new sterile petri dish, and the cultivation was shaken for 30 min at 37 °C. Centrifugation was performed at 2000 r/min for 15 min, and 20% serum concentration DMEM/F-12 cell culture medium was resuspended and then transferred to cell culture flask for culture. Then, PPMC were isolated from piglet and were cultivated in DMEM/F12 medium (Gibco, Gaithersburg, MD, USA), with 10% fetal bovine serum (Gibco, Gaithersburg, MD, USA) and additional antibiotic at 37 °C in a 5% CO_2_-humidified atmosphere. After PPMC were inoculated in cell culture plate for approximately 12 h, the cells were treated with GPS and baicalin.

### 4.4. Cell Apoptosis

The cell apoptosis experiments were performed using Annexin V-FITC apoptosis assay kit (Absin, Shanghai, China) following the manufacturer’s protocol. Briefly, PPMC were seeded in black 12-well culture plate and were cultured in a humidified atmosphere of 5% CO_2_ in air at 37 °C for 24 h. The cells were treated with baicalin and GPS. After treatment, the cells were harvested with trypsin solution without EDTA (Genom, Hangzhou, China), washed, and re-suspended in 300 μL binding buffer containing 5 μL Annexin V-FITC for 15 min in the dark. Then, 5 μL propidium iodide was added to the binding buffer for 5 min. Before determining the cells, add 200 μL of 1 × binding buffer. The stained samples were analysed using a Cytomics FC 500 Flow Cytometer (Beckman, Brea, CA, USA), and the results were analyzed by FlowJo software.

### 4.5. RNA Isolation and Reverse Transcription Quantitative Polymerase Chain Reaction (RT-qPCR)

Briefly, PPMC cells were seeded in 12 wells cell culture plates and cultured at 37 °C in a 5% CO_2_ humidified atmosphere. Then, the cells were treated and washed with the PBS. Total RNA was isolated on ice from the cells by Trizol (Takara, Dalian, China) according to the manufacturer’s instructions. The A260/A280 ratio was measured, and the agarose gel electrophoresis was carried out to ensure the purity and integrity of total RNA samples. One microgram of total RNA was immediately reversed transcribed to cDNA by the Reverse Transcription Kit following the manufacturer’s instructions (Takara, Dalian, China). The cDNA was amplified by RT-qPCR using a SYBR Premix Ex Taq (Takara, Dalian, China) according to the manufacturer’s instructions. Data from the reaction were collected and analyzed by complementary computer software. Relative quantification of gene expression was calculated using the 2^−^^△△Ct^ method and normalized to *β*-actin in each sample. All the primers used in this study are shown in Table 1.

### 4.6. Western Blot

Treated PPMC cells were lysed, broken, and the protein concentration was determined. Then, twenty micrograms of cellular proteins from each group were separated by 12% SDS-PAGE gel and electroblotted onto PVDF membranes (Millipore, Bedford, MA, USA). The blocking buffer (Beyotime, Shanghai, China) was utilized to block the membranes for one hour, and then the membranes were washed and incubated overnight at 4 °C with the primary antibody. TBST was utilized to wash the membranes for three times and the membranes were further incubated secondary anti-IgG antibody. After washing the membranes with TBST for three times, membranes were visualized with the DAB horseradish peroxidase color development kit (Beyotime, Shanghai, China).

### 4.7. Statistical Analysis

All the different groups of experimental data were compared to the control data using one-way analysis of variance (ANOVA). All the assays were conducted with three independent assays. Significant and extremely significant differences were indicated by *p* < 0.05 and *p* < 0.01, respectively.

## 5. Conclusions

In conclusion, our findings suggest that GPS infection can induce apoptosis of PPMC cells via activating the PKC-MAPK signaling pathway in peritonitis infection model, and baicalin inhibited the PKC-MAPK signaling pathway and alleviated the progress of cell apoptosis. Our study may provide a potential host defense mechanism against GPS and provide a possible therapeutic pathway for alleviating GPS infection, and baicalin could be a therapeutic option in the management of GPS infection.

## Figures and Tables

**Table 1:** Primer sequences for RT-qPCR.

Gene	Nucleotide Sequences (5’–3’)		T_m_ (°C)	Length (bp)
*β-actin*	Forward	TGCGGGACATCAAGGAGAAG	57.4	216
	Reverse	AGTTGAAGGTGGTCTCGTGG	57.4	
*Bax*	Forward	GCCGAAATGTTTGCTGACG	55.2	156
	Reverse	GAGCCGATCTCGAAGGAAGT	57.4	
*C-myc*	Forward	GGTCTTCCCCTACCCACT	57.2	200
	Reverse	CCTCATCCTCTTGTTCTTCC	55.4	
*Bcl-xl*	Forward	GCAACCCATCCTGGCACCT	59.5	136
	Reverse	TCAAACTCATCGCCCGCCT	57.3	
*c-fos*	Forward	GCTGACAGATACACTCCAAGCGG	61.3	542
	Reverse	AGGAAGACGTGTAAGTAGTGCAG	57.8	
*c-jun*	Forward	CGCCAGTCTACGCTAATC	55.1	288
	Reverse	GGTTCCTCATACGCTTCC	54.8	

## Data Availability

The data presented in this study are available on request from the corresponding author.
